# Clinical and Genetic Characteristics of Pheochromocytoma and Paraganglioma: A Single-Center Experience Including a Rare VHL Variant

**DOI:** 10.3390/jcm15020712

**Published:** 2026-01-15

**Authors:** Merve Korkmaz Yilmaz, Ozlem Kandemir Alibakan, Aydeniz Aydin Gumus, Alper Gezdirici, Huseyin Karatay, Serkan Sari, Tugba Matlim Ozel, Mutlu Niyazoglu, Esra Hatipoglu

**Affiliations:** 1 Department of Endocrinology, Basaksehir Cam and Sakura City Hospital, University of Health Sciences, 34480 Istanbul, Turkey; drmervekorkmaz@gmail.com (M.K.Y.);; 2 Department of Medical Genetics, Basaksehir Cam and Sakura City Hospital, University of Health Sciences, 34480 Istanbul, Turkey; 3 Department of Pathology, Basaksehir Cam and Sakura City Hospital, University of Health Sciences, 34480 Istanbul, Turkey; 4 Department of General Surgery, Basaksehir Cam and Sakura City Hospital, University of Health Sciences, 34480 Istanbul, Turkey

**Keywords:** pheochromocytoma, paraganglioma, germline mutation, *VHL*, *NF1*, rare variant

## Abstract

**Background/Objectives:** Advances in the genetic understanding of pheochromocytoma–paraganglioma (PPGL) have considerably refined personalized approaches to diagnosis and management. This study aims to present our institutional experience on the diagnostic characteristics, clinical course, and genetic background of patients with PPGL, in the context of the current literature. **Methods:** This retrospective analysis included 35 patients diagnosed with PPGL between years 2020 and 2024, all of whom underwent surgical resection and next-generation sequencing for germline mutations in major PPGL susceptibility genes. Clinical presentation, biochemical profile, pathological findings, and follow-up outcomes were compared between mutation-positive and mutation-negative cases. **Results:** Of the 35 patients with PPGL, germline mutations were identified in 6 patients (17%): 2 in Cluster 1A genes (*SDHA*, *SDHB*), 2 in Cluster 1B (*VHL*), and 2 in Cluster 2 (*NF1*). Consistent with existing literature, pathogenic germline variants—particularly *SDHB* and *VHL*—were identified in our cohort exclusively in patients younger than 30 years (ages 17, 20, and 25). Mutation-positive patients more frequently exhibited noradrenergic or non-secretory profiles (*p* = 0.01). Among the three non-secretory tumors in the cohort, two harbored genetic mutations (*SDHA*, *NF1*). Interestingly, both *NF1*-positive patients were normotensive—one (*c.3496G > A*) with a non-secretory tumor and the other (*c.2329T > A*) presenting at an unusually late age (63 years)—a strikingly atypical spectrum that underscores the phenotypic variability of *NF1*-associated PPGL. Bilateral disease was observed exclusively in *VHL* carriers (*p* = 0.03). Importantly, we identified a rare *VHL c.369delG* frameshift variant, not previously reported in association with PPGLs, in a patient with PPGL. No significant difference was observed between SDHB loss (*p* = 0.1) and proliferative indices (mitotic count, Ki-67) (*p* = 0.07, *p* = 0.6) between the two groups. During a median follow-up of 24 months (IQR: 18–36), one *SDHB*-positive patient had a recurrence, while no distant metastases were detected in the remaining mutation carriers. **Conclusions:** These findings support characteristic clinical patterns among mutation-positive PPGL and underscore the importance of systematic germline testing in all cases—irrespective of age, family history, or biochemical profile—to guide individualized management and enable cascade screening. The identification of a rare *VHL c.369delG* variant, previously unreported in association with PPGL, within a characteristic VHL-related clinical phenotype highlights the importance of this association. Similarly, atypical *NF1* cases emphasize phenotypic variability and reinforce the importance of germline testing even in clinically silent presentations.

## 1. Introduction

Pheochromocytomas and paragangliomas (PPGLs) are rare neuroendocrine tumors originating from chromaffin cells of the adrenal medulla (pheochromocytomas) or extra-adrenal sympathetic and parasympathetic paraganglia (paragangliomas). The incidence in the general population is approximately 0.8 per 100,000, and it is observed in 0.1–0.6% of individuals with hypertension [1]. Although PPGLs are considered a rare neoplasm, they can lead to severe and potentially fatal cardiovascular and metabolic complications.

The genetic landscape of PPGL has evolved rapidly over the past decade, leading to a significantly enhanced understanding of the disease process. Identifying new inherited forms of PPGL has revealed a germline susceptibility rate approaching 40–60%, one of the highest observed among all cancer types [2]. More than 22 susceptibility genes associated with PPGL have been identified. A strong genotype–phenotype correlation demonstrated across numerous studies frequently determines the clinical manifestations of syndromic forms of the disease [3]. These correlations influence various aspects, including biochemical profile, tumor localization, malignant potential, clinical aggressiveness, and overall prognosis. Furthermore, identifying the underlying genetic alteration is pivotal in treatment planning and provides essential guidance for appropriate follow-up and surveillance strategies [4].

PPGL-associated genes are classified into three major molecular clusters: Cluster 1 (Pseudohypoxia), subdivided into 1A and 1B, which includes genes involved in hypoxia-related pathways. Cluster 1A comprises genes encoding tricarboxylic acid (TCA) cycle enzymes, such as *SDHx*, *IDH1*, *FH*, *SUCLG2*, *MDH2*, and *GOT2*, all activating hypoxic signaling pathways. Cluster 1B encompasses mutations in genes such as *VHL*, *PHD1/2,* and *EPAS1* [5]. Cluster 2 (Kinase signaling): Includes genes such as *RET*, *NF1*, *HRAS, FGFR1*, *TMEM127*, and *MAX*, which activate kinase signaling cascades [6]. Cluster 3 (Wnt signaling): A more recently described group that includes rare mutations in *CSDE1* or *MAML3* fusion genes, which lead to Wnt pathway activation [7].

In this study, we aimed to characterize the clinical features of genetically associated PPGL managed at our institution

## 2. Methods

### 2.1. Study Design and Participants

This retrospective study included 35 patients diagnosed with PPGLs between years 2020 and 2024 at the Endocrinology Clinic of Basaksehir Cam and Sakura City Hospital. The study was approved by the local Clinical Research Ethics Committee (19 March 2025, decision no: 2025-107) and conducted in accordance with the Declaration of Helsinki.

All patients with a confirmed diagnosis underwent surgical treatment. Demographic, clinical, biochemical, imaging, genetic, and pathological data were collected from medical records. Plasma-free metanephrines and 24-h urinary fractionated metanephrines were measured. Plasma and 24-h urinary catecholamines and metanephrines were measured using liquid chromatography coupled with tandem mass spectrometry. Plasma-free metanephrines and 24-h urinary fractionated metanephrines were measured in all patients. In addition, 24-h urinary catecholamines, including dopamine, were assessed using liquid chromatography coupled with tandem mass spectrometry, performed on an Ultra Performance Liquid Chromatography (UPLC) system (Shimadzu, Kyoto, Japan) coupled with a SciEX QTRAP^®^ 3500 mass spectrometer (Sciex, Framingham, MA, USA). Methoxytyramine levels were not measured.

Additionally, pathological features including capsular and vascular invasion, lymph node and distant metastasis, periadrenal fat invasion, mitotic count, Ki-67 proliferation index, and SDHB immunohistochemical status were evaluated. SDHB immunohistochemistry was performed on formalin-fixed, paraffin-embedded tumor sections using a commercially available antibody. SDHB loss was defined as the absence of cytoplasmic staining in tumor cells with preserved staining in internal controls.

Genetic analysis was performed on all patients using next-generation sequencing (NGS) of DNA extracted from peripheral blood. Patients in whom no pathogenic variant was identified via NGS targeting PPGL-related genes were classified as genetically negative.

### 2.2. Genetic Testing

DNA isolated from the patients’ peripheral venous blood was sequenced using the Ion Torrent NGS method. A panel test including *SDHA*, *SDHB*, *SDHC*, *SDHD*, *SDHAF2*, *VHL*, *EPAS1*, *EGLN1*, *EGLN2*, *IDH1*, *FH*, *MDH2*, *NF1*, *MAX*, *RET*, *TMEM127*, *FGFR1*, *HRAS*, and *CSDE1* genes was performed, and the mean sequencing depth for each gene was greater than 90×. Pathogenic/likely pathogenic variants according to the American College of Medical Genetics (ACMG) 2015 scale were analyzed alongside the patients’ clinical findings. Clinically significant variants were confirmed by Sanger sequencing.

### 2.3. Statistical Analysis

Statistical analyses were performed using IBM SPSS Statistics for Windows, Version 25.0 (IBM Corp., Armonk, NY, USA). Descriptive statistics were presented as frequencies and percentages (n, %) for categorical variables, and as mean ± standard deviation (SD) or median (interquartile range, IQR) for continuous variables, as appropriate. The Mann–Whitney U test was used to compare two independent groups. Fisher’s exact test was applied for comparisons of categorical variables. A *p*-value of <0.05 was considered statistically significant. Given the limited number of mutation-positive cases, statistical findings were interpreted cautiously, and *p*-values were regarded as exploratory.

## 3. Results

### 3.1. Demographics and Clinical Characteristics

This study included 35 patients diagnosed with PPGLs, of whom 17% carried pathogenic germline variants. The proportion of females was similar across both groups: 55% in the mutation-negative group and 67% in the mutation-positive group, indicating no significant gender-based distribution difference (*p* = 0.7). The mean age in the mutation-positive group was 34.7 ± 17.9 years, which was younger than that in the mutation-negative group (45 ± 11.6 years); however, the difference was not statistically significant (*p* = 0.1). The youngest case was positive for the *SDHB* mutation and was identified at age 17. On the other hand, the eldest case was identified at age 63 and was positive for the *NF1* mutation.

Table 1 summarizes the characteristics of the cases based on hormonal and metabolic profiles. Mutation-positive patients exhibited a significantly higher frequency of noradrenergic (50%) and non-secretory (33%) phenotypes compared with the mutation-negative group (*p* = 0.01). On the other hand, the mutation-negative group predominantly displayed a mixed and noradrenergic phenotype (48%) (*p* = 0.01). Here, the term “mixed” was used descriptively to denote tumors that secrete both adrenaline (metanephrine) and noradrenaline (normetanephrine), in line with the commonly used biochemical descriptions in PPGL studies.

Hypertension was observed in 79% of patients in the mutation-negative group and in 50% of those with germline mutations (*p* = 0.2). Persistent hypertension was more frequent in the mutation-positive group (33% vs. 22%), while hypertensive crises were more common among mutation-negative cases (78% vs. 67%); however, neither difference reached statistical significance (*p* > 0.05). All hypertensive patients were receiving regular antihypertensive therapy, with a median treatment duration of 7 years. Preoperative blood pressure control was achieved using alpha-adrenergic blockade. Normotension was observed in 21% of mutation-negative patients and in 50% of those carrying pathogenic mutations (including two patients with *NF1* mutations and one with a *VHL* mutation).

In both groups, tumors were most frequently discovered incidentally during imaging, with MRI being the primary modality. All lesions identified on MRI displayed T2-weighted hyperintensity, consistent with classic radiological features of PPGL. Bilateral adrenal pheochromocytomas were observed in two patients, both of whom carried *VHL* mutations. The frequency of bilateral tumors was significantly higher in the mutation-positive group (*p* = 0.027). Adrenal localization was more prevalent in the mutation-negative group (97%) than in the mutation-positive group (67%), which included two extra-adrenal paragangliomas (*p* = 0.07). Tumor sizes were similar between groups. The majority of patients had tumors exceeding 4 cm in diameter: 83% in the mutation-positive group and 82% in the mutation-negative group. Among mutation-positive individuals, paragangliomas (60 mm and 130 mm) were generally larger than pheochromocytomas (ranging from 21 mm to 50 mm) (*p* = 0.1).

A comparable proportion of patients in both groups reported a family history of hypertension (51% vs. 49%, *p* > 0.05). Only one mutation-positive patient—a 20-year-old male with Von Hippel–Lindau syndrome—had a documented family history of pheochromocytoma.

### 3.2. Non-Secretory PPGL Cases

Non-secretory PPGL was defined based on repeatedly normal plasma-free metanephrine and 24 h urinary fractionated metanephrine measurements obtained under appropriate preanalytical conditions and in the absence of interfering medications. The first case involved a 35-year-old female who presented with episodes of hypertension and was found to have a 51 mm mass in the right adrenal gland. Genetic analysis revealed a heterozygous *SDHA c.466del* variant. In the second case, an abdominal CT of a 48-year-old woman with abdominal pain revealed a 130 × 124 mm complicated cystic lesion with thick walls, heterogeneous content, and prominent peripheral enhancement adjacent to the upper pole of the left kidney, displacing the kidney inferiorly and compressing the stomach. Genetic testing identified a heterozygous *NF1 c.3496G > A* variant, although no other clinical features of Neurofibromatosis type I were observed. The third case was a 44-year-old male with a 96 mm right adrenal mass and no detectable pathogenic mutations on genetic analysis. Notably, among the three non-secretory PPGL cases identified in the entire cohort, two harbored germline genetic variants.

### 3.3. Genetic, Pathological Findings and Follow-Up

Pathogenic germline mutations were identified in 6 of 35 patients (17%). Identified variants involved Cluster 1A genes (*SDHA* and *SDHB*), Cluster 1B genes (*VHL*), and Cluster 2 genes (*NF1*) (Table 2). Most variants had been previously reported in association with PPGL or related hereditary syndromes (Table 3).

The *SDHA c.466del* frameshift variant was identified in one patient and is absent from population databases such as gnomAD. This variant is listed in ClinVar as associated with PPGL syndrome 5 (ClinVar ID: 2452806) and is predicted to result in loss of protein function.

The *SDHB c.649C > G* missense variant was detected in one patient and is predicted by in silico analysis to affect protein function. This variant has been previously reported in association with PPGL.

The *VHL c.499C > T* (p.Arg167Trp) variant was identified in one patient and has been previously reported as pathogenic in association with von Hippel–Lindau disease. This variant is listed in ClinVar (Variation ID: 2218) and has been described in multiple affected families.

The *VHL c.369delG* (p.Thr124HisfsTer35) variant is predicted to result in a truncated protein and subsequent loss of *VHL* function. According to ClinVar, this variant is classified as pathogenic (Variation ID: 3148727; last accessed 8 January 2026). Although it has been reported once in ClinVar in a patient with von Hippel–Lindau disease, no detailed phenotypic information has been reported in the literature. This null variant, located in exon 2 of the *VHL* gene, affects a functional domain of the protein and is consistent with the established loss-of-function mechanism underlying VHL disease. In our cohort, the patient carrying the *VHL c.369delG* variant was a 20-year-old male with bilateral pheochromocytoma, a prior history of retinal hemangioblastoma, and a family history consistent with von Hippel–Lindau disease. To the best of our knowledge, this variant has not yet been reported in association with pheochromocytoma or paraganglioma. Accordingly, this finding represents a rare *VHL* variant previously unreported in association with PPGL, while the observed clinical presentation is consistent with the known *VHL*-related phenotype.

The *NF1 c.2329T > A* (p.W777R) variant was identified in one patient and has been previously reported as pathogenic in individuals with neurofibromatosis type 1.

The *NF1 c.3496G > A* (p.G1166S) variant was detected in one patient and is predicted to affect splicing. This rare variant has been previously reported in association with neurofibromatosis type 1 and has also been described in patients with PPGL.

Pathological features are summarized in Table 1. Capsular invasion was observed in 17% of mutation-positive cases and 9% of mutation-negative cases (*p* = 0.6), while vascular invasion was detected in 17% and 3% of cases, respectively (*p* = 0.3). No lymph node involvement or gross invasion was observed in either group.

Loss of SDHB expression on immunohistochemistry was observed in 33% (2/6) of mutation-positive tumors and 7% (2/29) of mutation-negative tumors, without a statistically significant difference between groups (*p* = 0.1). The Ki-67 proliferation index did not differ significantly between groups (*p* = 0.6), whereas mitotic count showed a non-significant trend toward higher values in mutation-positive cases (*p* = 0.07).

During a median follow-up of 24 months (IQR:18–36), recurrence was observed in one mutation-positive patient carrying an *SDHB* mutation and in one mutation-negative patient; all distant metastases were detected during follow-up (metachronous). One mutation-negative patient died due to causes unrelated to pheochromocytoma.

## 4. Discussion

In this study, we demonstrated that 17% of patients with PPGL carried germline mutations in *SDHA, SDHB, VHL,* and *NF1*. Mutation-positive cases exhibited clinical features, including a higher prevalence of noradrenergic or non-secretory phenotypes and an increased frequency of bilateral tumors, with bilateral disease observed exclusively in *VHL* carriers. Most importantly, we identified a rare *VHL c.369delG* variant, contributing to the expanding mutational spectrum of PPGL. Although SDHB loss and Ki-67 index did not differ significantly between mutation-positive and mutation-negative cases, our findings highlight the importance of routine germline testing to improve risk stratification and personalized management.

Current guidelines recommend that all patients diagnosed with PPGL undergo genetic evaluation [8]. Previous studies have demonstrated that germline mutations account for a substantial proportion of PPGL cases, with prevalence ranging from 10% to 30%, depending on patient selection and the scope of genetic testing panels [9]. Data on the germline mutational spectrum in the Turkish population are limited. Yalcintepe et al. conducted the first and largest targeted NGS study in Turkey on PPGL susceptibility genes, identifying pathogenic or likely pathogenic germline variants in 24% of 75 consecutively tested patients, comprising *RET* mutations in 6.7% and *non-RET* variants in *VHL, SDHB,* and *SDHD* in 17.3% [10]. This prevalence is lower than that reported in large Western cohorts—Italy (31%), the United States (27–31%), and the United Kingdom (30%)—and substantially lower than in some Asian series, where rates may reach 40% [5,11,12,13,14].

In our series, pathogenic variants were detected in 6 of 35 patients (17%): 2 in cluster 1A (*SDHA, SDHB*), 2 in cluster 1B (*VHL*), and 2 in cluster 2 (*NF1*). The relatively low detection rate is likely attributable to the limited cohort size and potentially lower mutation prevalence within the Turkish population. No *RET* mutations were identified, consistent with Turkish data [10] reporting *RET* and *non-RET* mutation rates of 6.7% and 17.3%, respectively. Similar patterns have been reported in other populations, with *non-RET* mutations predominating in a Saudi Arabian multicenter cohort (35.5%) and a Korean cohort (27.8%) [15,16]. These findings suggest a consistently higher prevalence of *non-RET* mutations across diverse populations, with overall detection rates influenced by both population-specific variation and sample size.

Among the six pathogenic or likely pathogenic germline variants identified in our cohort, five have been previously reported to be associated with PPGL or related syndromes. In contrast, the *VHL c.369delG* (p.Thr124HisfsTer35) frameshift variant, while listed once in ClinVar (ClinVar ID: 3148727) in a patient with Von Hippel–Lindau disease, has not been described in detail in the literature and, to our knowledge, has not previously been reported in association with pheochromocytoma. This loss-of-function alteration, located in exon two and affecting a functional domain of the *VHL* protein, is consistent with the established tumorigenic mechanism of VHL disease. Its identification in our patient represents a rare *VHL* variant previously unreported in association with PPGL, expanding the mutational spectrum of *VHL*-related PPGL and underscoring the importance of systematic genetic testing in all patients.

PPGLs are most frequently diagnosed in the fourth and fifth decades of life [17]. The mean age in the mutation-positive group was 34.7 ± 17.9 years, which was younger than that in the mutation-negative group (45 ± 11.6 years); however, the difference was not statistically significant (*p* = 0.1). Compared with mutation-negative cases, pathogenic germline variants, particularly *SDHB* and *VHL*, are more often found in patients under 30 years old. Consistent with existing literature, our patients with *VHL* mutations were aged 20 and 25 years, while those with an *SDHB* mutation were 17 years old (<30 years). Interestingly, one patient with an *NF1*-associated variant was diagnosed at 63 years—well beyond the usual age of presentation for *NF1*-PPGL, which averages 42–46 years [18]. Moreover, this patient lacked other classical NF1 manifestations, underscoring phenotypic variability and the possibility of late-onset PPGL even in *NF1* mutation carriers. These observations highlight that while *NF1*-associated PPGL generally presents in middle age, exceptions do occur—and absence of typical NF1 features should not preclude genetic evaluation or vigilant surveillance. In both *NF1* mutation carriers, a systematic clinical evaluation was performed, including dermatological examination, ophthalmologic assessment, and detailed family history review, and no additional NF1-related clinical features were identified at the time of diagnosis.

Hypertension was present in 79% of mutation-negative cases and in 50% of mutation-positive patients in our cohort. Persistent hypertension occurred more frequently in the mutation-positive group (33% vs. 22%), whereas hypertensive crises were more common in the mutation-negative group (78% vs. 67%). Among mutation-positive hypertensive patients, germline variants were identified in *VHL*, *SDHA*, and *SDHB*. In the literature, hypertension is reported in the majority of PPGL cases regardless of genetic status; however, several studies suggest genotype-specific differences in blood pressure phenotype. Notably, tumors associated with pseudohypoxia pathway genes such as *VHL* and *SDHx* are typically characterized by lower catecholamine secretion and a higher likelihood of normotension. In contrast, our series identified hypertensive presentations in patients harboring *VHL, SDHA,* and *SDHB* variants, underscoring the heterogeneity of blood pressure phenotypes even within pseudohypoxia-related genotypes [2,18].

In our study, normotensive patients—defined as having sustained blood pressures below 140/90 mmHg without antihypertensive therapy—accounted for 50% of the mutation-positive group (two *NF1* and one *VHL*) and 21% of the mutation-negative cases. The occurrence of normotension in our *VHL*-positive patient is consistent with reports linking *VHL* mutations to lower catecholamine production and milder cardiovascular manifestations [19,20]. Conversely, the presence of normotension in two *NF1*-positive patients (harboring the *c.3496G > A* and *c.2329T > A* variants) is less typical, as *NF1*-associated PPGLs are generally adrenergic and hypertensive. However, normotensive presentations have been described, particularly in small or incidentally discovered tumors [21,22].

A family history of hypertension was comparable between groups (50% vs. 52%), suggesting limited predictive value for distinguishing hereditary from mutation-negative PPGL, consistent with prior observations that essential hypertension is common in the general population and not specific to PPGL predisposition [23]. Collectively, these findings highlight that while specific genotypes may influence the likelihood and phenotype of hypertension, the absence of hypertension—particularly in mutation carriers—does not exclude the diagnosis and should not delay biochemical or genetic evaluation.

A family history of pheochromocytoma was identified in only one patient with VHL syndrome. This finding may be attributable to incomplete uptake of recommended family screening, the presence of clinically silent cases, or de novo mutations in the proband. More broadly, an explanation for the high frequency of hereditary pheochromocytoma without a family history of disease might include spontaneous mutation in a susceptibility gene, decreased penetrance, and maternal imprinting. Reduced penetrance has been documented in multiple PPGL-associated genes, including *SDHB*, *SDHD,* and *TMEM12*7, leading to delayed or absent disease manifestation in mutation carriers [24]. Maternal imprinting, particularly relevant for *SDHD* and *SDHAF2*, results in pathogenic variants being phenotypically expressed only when paternally inherited, further complicating pedigree-based risk assessment [25]. These factors highlight the limitations of relying solely on family history to guide genetic testing and underscore the necessity for systematic germline evaluation in all patients with PPGL.

Mutation-positive patients exhibited a significantly higher frequency of noradrenergic (50%) and non-secretory (33%) phenotypes (*p* = 0.014). In contrast, the mutation-negative group predominantly displayed a mixed and noradrenergic phenotype (48%) (*p* = 0.014). In this study, the term “mixed” was used descriptively to indicate tumors with combined adrenaline and noradrenaline secretion and was not intended to define a distinct secretory phenotype. These findings are consistent with previous reports indicating that germline mutations—particularly in pseudohypoxia pathway genes (e.g., *VHL, SDHx*)—are frequently associated with a noradrenergic secretory profile or biochemical silence, reflecting lower overall catecholamine output [5,26]. Conversely, mutation-negative PPGLs and tumors linked to kinase signaling pathway genes (e.g., *RET*, some *NF1* variants) are more likely to display mixed or predominantly adrenergic secretion, correlating with higher circulating epinephrine levels and more symptomatic presentations [6]. The relatively high proportion of non-secretory tumors in our mutation-positive subgroup—particularly in patients harboring *SDHA* and *NF1* variants—is noteworthy, as non-secretory presentations have been reported more frequently in cluster 2 mutations or head and neck paragangliomas, but less commonly in adrenal pheochromocytomas. This biochemical phenotype may partially explain the lower prevalence of hypertensive crises among mutation carriers in our series. Given the statistically significant difference observed, these results support the notion that the underlying genotype exerts a substantial influence on catecholamine metabolism and release. Larger, multi-center studies are warranted to validate these findings and to clarify their implications for genotype-informed biochemical screening and surveillance strategies.

The term “non-secretory PPGL” is preferred over “clinically silent” to describe tumors that lack catecholamine secretion, as confirmed by repeated plasma or 24-h urine tests [27]. Prior studies have suggested that non-secretory PPGLs are more frequently associated with cluster 2 mutations, often demonstrating low plasma catecholamine output despite histologically adrenergic features. In our series, three patients (9%) had non-secretory PPGLs, two of whom harbored germline mutations—*SDHA c.466del* and *NF1 c.3496G > A*. The patient carrying the *SDHA c.466del* variant was referred to the endocrinology outpatient clinic for evaluation of an adrenal incidentaloma and paroxysmal hypertension. Biochemical testing revealed persistently normal plasma and urinary catecholamine levels. Imaging demonstrated a unilateral 50-mm adrenal mass, and given the absence of a history of malignancy but a lesion size exceeding 4 cm, surgical resection was performed. Histopathological examination confirmed the diagnosis of pheochromocytoma. *SDHA*-positive PPGLs span a wide age range (11–81 years), affect both sexes equally, and are often solitary tumors in the head/neck or abdomen. Metastases occur in 25% of cases, and familial history is rare [28]. Bilateral presentation is not uncommon in hereditary syndromes, including those involving *SDH* mutations. Tumors with *SDHA* mutations may exhibit a noradrenergic phenotype. Unilateral, non-secretory pheochromocytoma in our patient with an *SDHA c.466del* mutation is a remarkable feature. The patient harboring the *NF1 c.3496G > A* variant was referred for evaluation of an incidentally detected large abdominal mass. She had no personal or family history suggestive of neurofibromatosis type 1 and exhibited no cutaneous or neurological stigmata of the disease. Biochemical testing confirmed normal plasma and urinary catecholamine levels. Cross-sectional imaging revealed a unilateral abdominal paraganglioma measuring 130 mm, and surgical excision was undertaken. Histopathology was consistent with paraganglioma. While PPGLs in *NF1* typically exhibit an adrenergic secretory profile and are present in 0.1–5.7% of *NF1* cases by age 40, up to 30% are incidentally detected, and fewer than 20% are symptomatic [29,30]. This atypical presentation—characterized by an exceptionally large, non-secretory paraganglioma in an *NF1* mutation carrier without classical phenotypic features—provides additional clinical insight into the spectrum of *NF1*-associated PPGL and underscores the importance of comprehensive genetic testing even in the absence of hallmark *NF1* manifestations. Taken together, these two mutation-positive, non-secretory cases—one *SDHA*-related and the other *NF1*-associated—represent rare biochemical phenotypes within their respective genotypes, thereby contributing additional clinical observations to the existing literature and reinforcing the need for genotype-driven diagnostic vigilance.

In our study, 83% (5/6) of mutation-positive and 82% of mutation-negative patients had tumors larger than 4 cm, consistent with previous reports showing no significant size difference between mutation carriers and non-carriers. However, within the mutation-positive subgroup, paragangliomas were larger at diagnosis than pheochromocytomas (60–130 mm vs. 21–50 mm). This finding aligns with the literature, which indicates that extra-adrenal paragangliomas often exhibit reduced or absent catecholamine secretion, particularly in tumors with a noradrenergic or non-secretory biochemical phenotype, leading to attenuated symptomatology and delayed clinical detection [26]. Several studies have shown that the time to diagnosis for paragangliomas is frequently prolonged compared to adrenal pheochromocytomas, with tumor size at presentation reflecting diagnostic latency rather than intrinsic proliferative capacity. Longitudinal volumetric assessments have estimated median tumor doubling times of approximately 5–7 years for PPGLs, supporting the notion that indolent growth combined with low biochemical activity contributes to their often substantial size at diagnosis. Notably, in our cohort, both large paragangliomas occurred in patients harboring germline mutations (*SDHB* and *NF1*), a pattern consistent with literature linking hereditary PPGL syndromes to a higher likelihood of extra-adrenal location, non-secretory phenotype, and delayed clinical recognition [2,5]. This observation underscores the need for structured biochemical and imaging surveillance in genetically predisposed individuals, regardless of symptomatic status, to prevent the late detection of clinically silent but potentially large lesions.

The frequency of bilateral tumors was significantly higher in the mutation-positive group (*p* = 0.027). Bilateral adrenal pheochromocytomas were observed in two patients, both of whom carried *VHL* mutations. This finding reinforces the well-established association between *VHL* mutations and bilateral adrenal involvement. In individuals with *VHL* gene mutations, PPGL frequently develops before the age of 20 and is bilateral in 40–60% of cases, either synchronously or metachronously [31]. Approximately two-thirds of such patients present with a family history of VHL disease or associated syndromic manifestations [32]. In our series, the first *VHL*-positive case was a 20-year-old male with a prior history of retinal hemangioblastoma surgery, in whom bilateral adrenal masses (22 mm and 13 mm) were detected. A family history of pheochromocytoma was present, and genetic testing confirmed a pathogenic *VHL* variant. Two siblings of this patient carried the same mutation and were diagnosed with retinal hemangioblastoma, while a cousin was found to have *VHL*-positive renal cell carcinoma. The second *VHL* case involved a 25-year-old female investigated for secondary hypertension, with plasma normetanephrine levels 10-fold above the upper limit of normal. Imaging revealed bilateral adrenal masses (40 mm and 15 mm), and bilateral adrenalectomy was performed. Subsequent systemic screening for VHL revealed a 1 cm right cerebellar hemangioblastoma on brain MRI, for which neurosurgical follow-up was advised. These observations underscore that, in the context of a hereditary PPGL syndrome—particularly *VHL*—the presence of a tumor in one adrenal gland should prompt systematic contralateral evaluation, even in the absence of clinical or biochemical suspicion. This is critical not only for surgical planning but also for family screening and surveillance strategies. The bilateral presentation observed in our *VHL*-positive cases is consistent with prior literature and reinforces the value of comprehensive genetic assessment in all PPGL patients.

In our cohort, no significant differences were observed between the two groups for capsular invasion, adipose tissue invasion, vascular invasion, periadrenal fat invasion, Ki-67 proliferative index, and mitosis count. Though part of PASS and GAPP scores, these features alone poorly predict metastasis [33,34]. Their prognostic value increases when combined with proliferation, biochemical, and genetic data, especially in high-risk genotypes like *SDHB*. On the other hand, SDHB loss was detected in 33.3% (2/6) of mutation-positive patients, compared with 6.9% (2/29) in mutation-negative patients (*p* = 0.12). Among genetically positive cases, SDHB loss occurred in patients carrying *SDHA* or *SDHB* mutations. *SDHB* encodes a critical subunit of mitochondrial complex II, facilitating electron transfer within the respiratory chain by catalyzing the oxidation of succinate to fumarate in the tricarboxylic acid cycle [35]. Loss of *SDHB* function leads to succinate accumulation, inhibition of prolyl hydroxylases, and stabilization of hypoxia-inducible factors, thereby activating pseudohypoxic signaling pathways implicated in tumorigenesis [36]. Although classically associated with germline *SDHB* variants, SDHB loss is not exclusive to them and may reflect somatic *SDHx* alterations, promoter methylation, or secondary mitochondrial dysfunction in sporadic cases. SDHB loss has been linked to a higher risk of malignancy in pheochromocytomas and sympathetic paragangliomas [37]. In our cohort, one mutation-positive patient with SDHB loss developed distant metastases during follow-up. This finding aligns with the current literature, which suggests that SDHB immunohistochemistry may serve as a prognostic tool to guide surveillance intensity, particularly in high-risk individuals.

In our cohort, the median follow-up duration was 24 months. Postoperative hypertension persisted in seven patients, all of whom belonged to the mutation-negative group. None of the mutation-positive patients exhibited persistent hypertension, achieving complete biochemical remission and normotension following surgery. This finding aligns with previous reports suggesting that mutation-positive PPGL—particularly those involving pseudohypoxia pathway genes such as *VHL* and *SDHx*—often exhibit lower catecholamine output, which may facilitate normalization of blood pressure after tumor resection [18,19,20]. A mutation-negative patient died from causes unrelated to pheochromocytoma. Recurrence occurred in one mutation-negative and one *SDHB*-mutated case. A 17-year-old female patient with an *SDHB* mutation initially diagnosed as a noradrenergic abdominal paraganglioma experienced recurrence of a pre-aortic mass three years after resection. *SDHB* germline mutations are key predictors of metastatic PPGL, with reported rates of 50–90% [38]. While malignant progression is not inevitable and may vary by variant, this case reflects the recognized aggressiveness of *SDHB*-related tumors [39]. It highlights the need for lifelong surveillance, regardless of the initial stage. *SDHB* mutations primarily predispose to abdominal paragangliomas (60%), with a median age of diagnosis below 30 years, and 50% penetrance by age 50 years [40,41]. In our series, cascade testing revealed the same *SDHB* variant in the patient’s mother and two siblings, all currently asymptomatic. Given the risk of late-onset disease, structured follow-up with annual biochemical testing and periodic whole-body imaging is essential for affected individuals.

This study is not without certain limitations. The small sample size and retrospective design may limit the generalizability of our findings. In addition, tumor tissue sequencing was not performed, which precludes direct assessment of somatic alterations and limits the interpretation of clinical patterns based solely on germline genetic findings. Furthermore, the relatively short median follow-up period may limit evaluation of long-term outcomes, especially for high-risk genotypes including *SDHB*. Genetic and clinical variations may differ across populations, highlighting the need for further research to develop ethnicity-specific genetic testing algorithms tailored to the Turkish population. Expanding our understanding of the molecular pathogenesis of PPGL is essential to improve diagnostic accuracy, enhance surveillance strategies, and support the development of targeted therapies for these rare tumors.

In summary, our findings underscore the clinical and genetic heterogeneity of PPGL and support the routine implementation of germline genetic testing. The identification of rare, previously unreported variants, such as *VHL* c.369delG, underscores the importance of expanding mutational databases and conducting population-specific studies. While our cohort size was limited, the observed clinical patterns associated with specific genetic findings contribute to a growing body of evidence informing individualized management strategies. Continued research, including larger multicenter studies, is essential to refine risk-stratification models, guide surveillance protocols, and ultimately improve outcomes in patients with hereditary and sporadic PPGL.

## 5. Conclusions

This study demonstrates the substantial genetic and clinical heterogeneity of pheochromocytoma and paraganglioma and reinforces the importance of systematic germline testing in all patients. Distinct features observed in mutation-positive cases highlight the need for genotype-informed surveillance strategies. Integration of molecular diagnostics into routine practice may improve risk stratification and long-term outcomes in PPGL.

## Figures and Tables

**Table 1:** Demographic and clinical characteristics of the patients.

Variables	All Patients (n = 35)	Mutation Positive (n = 6)	Mutation Negative (n = 29)	*p*-Value
Age				
Years (mean ± SD)	43.3 ± 13.2	34.7 ± 17.9	45 ± 11.6	0.1 ^a^
Gender				
Female, n (%)	20 (57%)	4 (67%)	16 (55%)	0.7 ^b^
Male, n (%)	15 (43%)	2 (33%)	13 (45%)	
Hypertension				
Yes, n (%)	26 (74%)	3 (50%)	23 (79%)	0.2 ^b^
No, n (%)	9 (26%)	3 (50%)	6 (21%)	
Hypertension type				
Attacks, n (%)	20 (57%)	2 (33%)	18 (62%)	1.0 ^b^
Continuous, n (%)	6 (17%)	1 (17%)	5 (17%)	
Family history of hypertension				
Yes, n (%)	18 (51%)	3 (50%)	15 (52%)	1.0 ^b^
No, n (%)	17 (49%)	3 (50%)	14 (48%)	
Family history of pheochromocytoma				
Yes, n (%)	1 (3%)	1 (17%)	0 (0)	0.2 ^b^
No, n (%)	34 (97%)	5 (83%)	29 (100%)	
PPGL secretory profile				
Mixed, n (%)	14 (40%)	0 (0)	14 (48%)	0.01 ^b^
Noradrenergic, n (%)	16 (45.7%)	3 (50%)	13 (44%)	
Adrenergic, n (%)	2 (5.7%)	1 (17%)	1 (4%)	
Dopaminergic, n (%)	0 (0)	0 (0)	0 (0)	
Non-secretory, n (%)	3 (8.6%)	2 (33%)	1 (4%)	
Mass detection				
Incidental, n (%)	28 (80%)	4 (67%)	24 (83%)	0.6 ^b^
Non-incidental, n (%)	7 (20%)	2 (33%)	5 (17%)	
Imaging method				
CT, n (%)	6 (17%)	1 (17%)	5 (17%)	1.0 ^b^
MR, n (%)	29 (83%)	5 (83%)	24 (83%)	
Localization				
Adrenal, n (%)	32 (91%)	4 (67%)	28 (97%)	0.07 ^b^
Extra-adrenal, n (%)	3 (9%)	2 (33%)	1 (3%)	
Side localization (all PPGLs)				
Right, n (%)	21 (60%)	3 (50%)	18 (62%)	0.03 ^b^
Left, n (%)	12 (34%)	1 (17%)	11 (38%)	
Bilateral, n (%)	2 (6%)	2 (33%)	0 (0)	
Lesion size (cm)				
≤4 cm, n (%)	7 (20%)	1 (17%)	6 (21%)	1.0 ^b^
4–6 cm, n (%)	15 (43%)	3 (50%)	12 (41%)	
≥6 cm, n (%)	13 (37%)	2 (33%)	11 (38%)	
Capsule invasion				
Yes, n (%)	10 (29%)	1 (17%)	9 (31%)	0.6 ^b^
No, n (%)	25 (71%)	5 (83%)	20 (69%)	
Vascular invasion				
Yes, n (%)	2 (6%)	1 (17%)	1 (3%)	0.3 ^b^
No, n(%)	33 (94%)	5 (83%)	28 (97%)	
Lymph node invasion				
Yes, n (%)	0 (0)	0 (0)	0 (0)	1.0 ^b^
No, n (%)	35 (100%)	6 (100%)	29 (100%)	
Periadrenal fat tissue invasion				
Yes, n (%)	3 (8%)	0 (0)	3 (10%)	1.0 ^b^
No, n (%)	32 (92%)	6 (100%)	26 (90%)	
Presence of gross invasion				
Yes, n (%)	0 (0)	0 (0)	0 (0)	1.0 ^b^
No, n (%)	35 (100%)	6 (100%)	29 (100%)	
Distant metastasis				
Yes, n (%)	2 (6%)	1 (17%)	1 (3%)	0.3 ^b^
No, n (%)	33 (94%)	5 (83%)	28 (97%)	
Timing of metastasis				
At diagnosis (synchronous), n (%)	0 (0)	0 (0)	0 (0)	NA
During follow-up (metachronous), n (%)	2 (100%)	1 (100%)	1 (100%)	
SDHB loss				
Yes, n (%)	4 (11%)	2 (33%)	2 (7%)	0.1 ^b^
No, n (%)	31 (89%)	4 (67%)	27 (93%)	
Ki-67 (MIB %), median (IQR)	2.0 (1.0–2.0)	2.0 (1.3–2.8)	2.0 (1.0–2.0)	0.6 ^a^
Mitosis (2 mm^2^), median (IQR)	1.0 (1.0–2.0)	3.0 (2.3–3.0)	1.0 (1.0–3.0)	0.07 ^a^
Postoperative hypertension				
Yes, n (%)	7 (20%)	0 (0)	7 (25%)	0.3 ^b^
No, n (%)	28 (80%)	6 (100%)	22 (75%)	

^a^: Mann–Whitney U test, ^b^: Fisher’s exact test. *p* < 0.05 statistically significant, NA: not applicable due to the small number of events.

**Table 2:** Summary of the phenotype of genetic PPGL.

Gene Mutation	Age (yrs)	Gender	Localization	Uni/Bilateral	Tumor Size (mm)	Secretory Profile
*SDHA* c.466del	35	Female	Pheochromocytoma	Unilateral	50	Non-secretory
*SDHB* c.649C > G	17	Female	Abdominal paraganglioma	Unilateral	60	Noradrenergic
*VHL* c.499C > T	25	Female	Pheochromocytoma	Bilateral	41	Noradrenergic
*VHL* c.369delG	20	Male	Pheochromocytoma	Bilateral	20	Noradrenergic
*NF1* c.2329T > A	63	Male	Pheochromocytoma	Unilateral	50	Adrenergic
*NF1* c.3496G > A	48	Female	Abdominal paraganglioma	Unilateral	130	Non-secretory

**Table 3:** Types of neuroendocrine tumors with which the identified variants were previously associated, ACMG: American College of Medical Genetics.

Gene Mutation	Variant Type	ACMG Classification	Mutation-Associated Disease
*SDHA* c.466del (p.Tyr156Metfs70) (NM_004168.4) heterozygous	Frameshift	Pathogenic	Pheochromocytoma/Paraganglioma Syndrome 5
*SDHB* c.649C > G (p.Arg217Gly) (NM_003000.3) heterozygous	Missense	Pathogenic	Paraganglioma Syndrome 4
*VHL* c.499C > T (p.Arg167Trp) (NM_000551.4) heterozygous	Missense	Pathogenic	Pheochromocytoma and Paraganglioma
*VHL* c.369delG (p.Thr124HisfsTer35) heterozygous	Frameshift	Pathogenic	Von-Hippel Lindau Syndrome
*NF1* c.2329T > A (p.Trp777Arg) (NM_001042492.3) heterozygous	Missense	Pathogenic	Pheochromocytoma
*NF1* c.3496G > A (p.Gly1166Ser) (NM_001042492.3) heterozygous	Missense	Pathogenic	Pheochromocytoma

## Data Availability

The data are available from the corresponding author upon reasonable request.
